# Greenhouses represent an important evolutionary niche for *Alternaria alternata*

**DOI:** 10.1128/spectrum.00390-24

**Published:** 2024-05-10

**Authors:** Guangzhu Yang, Sai Cui, Wenjing Huang, Shutong Wang, Jun Ma, Ying Zhang, Jianping Xu

**Affiliations:** 1 State Key Laboratory for Conservation and Utilization of Bio-Resources in Yunnan, Yunnan University, Kunming, Yunnan, China; 2 College of Life Science, Yunnan University, Kunming, Yunnan, China; 3 Horticultural Research Institute, Yunnan Academy of Agricultural Sciences, Kunming, Yunnan, China; 4 College of Plant Protection, Hebei Agricultural University, Baoding, Hebei, China; 5 Department of Biology, McMaster University, Hamilton, Ontario, Canada; Universidade de Sao Paulo, Ribeirao Preto, Sao Paulo, Brazil

**Keywords:** fungal pathogen, *Alternaria*, fungicide tolerance, triazoles, population genetics, *cyp51*, metapopulation

## Abstract

*Alternaria alternata* is a ubiquitous soil-borne fungus capable of causing diseases in a variety of plants and occasionally in humans. While populations of *A. alternata* from infected plants have received significant attention, relatively little is known about its soil populations, including its population genetic structure and antifungal susceptibilities. In addition, over the last two decades, greenhouses have become increasingly important for food and ornamental plant production throughout the world, but how greenhouses might impact microbial pathogens such as *A. alternata* populations remains largely unknown. Different from open crop fields, greenhouses are often more intensively cultivated, with each greenhouse being a relatively small and isolated space where temperature and humidity are higher than surrounding environments. Previous studies have shown that greenhouse populations of two common molds, *Aspergillus fumigatus* and *A. alternata,* within a small community in southwestern China were variably differentiated. However, the relative contribution of physical separation among local greenhouses to the large-scale population structure remains unknown. Here, we isolated strains of *A. alternata* from seven greenhouses in Shijiazhuang, northeast China. Their genetic diversity and triazole susceptibilities were analyzed and compared with each other and with 242 isolates from nine greenhouses in Kunming, southwest China. Results showed that the isolation of greenhouses located <1 km from each other locally contributed similarly to the overall genetic variation as that between the two distant geographic regions. In addition, our results indicate that greenhouses could be significant sources of triazole resistance, with greenhouses often differing in their frequencies of resistant strains to different triazoles.

**IMPORTANCE:**

Greenhouses have become increasingly important for food production and food security. However, our understanding of how greenhouses may contribute to genetic variations in soil microbial populations is very limited. In this study, we obtained and analyzed soil populations of the cosmopolitan fungal pathogen *Alternaria alternata* in seven greenhouses in Shijiazhuang, northeast China. Our analyses revealed high proportions of isolates being resistant to agricultural triazole fungicides and medical triazole drugs, including cross-resistance to both groups of triazoles. In addition, we found that greenhouse populations of *A. alternata* located within a few kilometers showed similar levels of genetic differentiation as those separated by over 2,000 km between northeast and southwest China. Our study suggests that greenhouse populations of this and potentially other fungal pathogens represent an important ecological niche and an emerging threat to food security and human health.

## INTRODUCTION

*Alternaria alternata* is a ubiquitous mold and a cosmopolitan plant pathogen that can cause a range of diseases such as leaf blight on many economically important plants, including cereals, ornamental plants, edible oil crops, vegetables, and fruits (1, 2). In addition, latent infections on plants can occur and result in post-harvest diseases, damping-off, and black spots in the case of infected seeds and fruits (3). Furthermore, *A. alternata* can produce toxins such as alternariol, alternariol methyl ether, and altenuene, and these toxins can accumulate in maturing seeds, fruits, nuts of many crops, and their processed food products (4, 5). Contaminated foods can enter the food chain and cause severe health problems for consumers. While rare, *A. alternata* is increasingly recognized as an opportunistic human pathogen, especially in immunocompromised patients (1, 6, 7). The human diseases caused by *A. alternata* included hypersensitivity pneumonitis, asthma, keratitis, allergic fungal rhinitis and sinusitis, and cutaneous infections (8–10). Aside from being a pathogen, *A. alternata* is also a common saprophyte, consuming dead organic matter commonly found in soil. Indeed, the soil population likely represents the reservoir from where infecting propagules originate to cause diseases in plants and humans. Thus, to effectively control and prevent *A. alternata* infections in both agriculture and human hosts, we need a comprehensive understanding of its ecology and evolution in soil populations of this species (11, 12).

It is reported that globally, up to 2021, over 3.64 million hectares of greenhouses have been constructed (13). Over the last two decades, protective agriculture such as greenhouses has developed rapidly due to its controllability and efficiencies in land and energy utilization, especially in areas near metropolitan cities (14). However, agricultural greenhouses provide not only an ideal environment for vegetables, fruits, and ornamental plants but also conditions such as moderate to high temperature and high relative humidity that are also favorable for the growth of soil fungi, including plant and soil-borne pathogens (15). For instance, fungal diseases such as black mold caused by *A. alternata* on tomatoes and strawberries (16, 17), and downy mildew (by *Peronospora sparsa*), botrytis (by *Botrytics cinerea*), and powdery mildew (by *Sphaerotheca pannosa*) on cucumber, grape, strawberry, and other crops are commonly reported in greenhouses (14). Although integrative agronomic measures and precautions are recommended and implemented for controlling fungal diseases in greenhouses, farmers often rely on fungicides to control pathogens to maintain or enhance productivity and reduce disease damage (18, 19). Due to increasing evidence of environmentally acquired drug resistance spreading to human pathogen populations (20–22), it is extremely important to understand the ecological niches such as greenhouses in the development of drug-resistant genotypes of fungal pathogens such as *A. alternata*.

Indeed, due to the increasing use of agricultural fungicides, *A. alternata* field isolates resistant to one or more classes of fungicides have been discovered in various crops worldwide (23, 24). For example, a previous study reported that all *A. alternata* isolates from a Minneola tangelo orchard with 3 years of iprodione application history were resistant to iprodione (25). Similarly, strains of *A. alternata* and several other *Alternaria* species from several geographic regions showed medium to high resistance to site-specific fungicides such as quinone outside inhibitor and succinate dehydrogenase inhibitors (26–31). Decreased susceptibility to demethylation inhibitors (DMIs) has also been observed in this cosmopolitan pathogen. For example, *A. alternata* isolates with even limited exposure to difenoconazole (DIF), propiconazole, or metconazole from a pistachio orchard in California, USA, were found to be less sensitive to these triazoles than those without exposure history (32). Variable proportions of *A. alternata* isolates from potato fields in China were also found tolerant to DIF (24). In clinical practice, although cases are very limited, an *A. alternata* strain isolated from an alternariosis patient clinically not responsive to itraconazole showed an elevated MIC (2 mg/L) for itraconazole (9). For resistance to DMIs in *A. alternata*, a mutation of R511W at the *cyp51* gene and a 6-bp insertion in the promoter region of the *cyp51* gene were observed in some of the resistant isolates (24).

The primary dispersal method of species of *Alternaria* is likely through the release of conidia (asexual spores) into the air and spread by air currents (8). Both natural dispersal, such as by wind, and human-mediated movements, such as local to global trade of agricultural products, can facilitate the dispersal of this fungus (33). For pathogens in greenhouses, the greenhouse walls could be a potential barrier for spore dispersals, potentially contributing to genetic differentiation among greenhouse populations. Indeed, our recent analyses showed that most geographically closely located greenhouse soil populations of *Aspergillus fumigatus* and *A. alternata* within a small community in southwestern China showed variable genetic differences (21). Whether such relationships can be extended to other climate conditions and/or to greenhouse populations separated by long distances remains unknown. Understanding pathogen population structure and the geographical and ecological factors influencing their structure is critical to formulating effective and sustainable plant disease management strategies and to control and prevent the spread of fungicide-resistant pathogens.

In our recent population genetic analysis of *A. alternata* from greenhouse soils in metropolitan Kunming, Yunnan, southwest China, greenhouse structures were found to be a significant barrier for gene flow among local soil *A. alternata* populations (21). Those greenhouses were located at high altitude (>1,900 m above sea level) in a subtropical environment, where the climate was dominated by cool dry winters and mild monsoon summers. To study the relative importance of isolation by greenhouses to other evolutionary forces such as long-distance geographic separation, we analyzed *A. alternata* from seven greenhouses in Shijiazhuang, northeast China, located about 2,300 km from Kunming, with a very different climate and lower elevation (<100 m above sea level). We hypothesized that the different climates between Shijiazhuang in northeast China and Kunming in southwest China have likely led to different relationships among strains and populations of *A. alternata* among greenhouses within each region. Although Shijiazhuang and Kunming are located far apart from each other and with different crops, we also hypothesize that greenhouse crops will likely have limited effects on the soil *A. alternata* populations in greenhouses. Finally, given the frequent use of triazole fungicides in most greenhouses, we expect that the Shijiazhuang greenhouse populations will contain azole-resistant *A. alternata* strains, with frequencies of drug resistance similar to those in southwestern China. However, due to the large geographic distance between Kunming and Shijiazhuang, we also expect the genetic mutations associated with triazole resistance to have originated independently and be different between the two regions. To test these predictions, we isolated 233 *A*. *alternata* strains from seven greenhouses near Shijiazhuang, obtained their genotype and antifungal susceptibility information, and compared the genotypic and phenotypic variations among the seven greenhouses and with greenhouses from Kunming, southwest China.

## MATERIALS AND METHODS

### Isolate collection and identification

A total of 700 soil samples were collected from seven greenhouses located in Gaocheng, part of metropolitan Shijiazhuang, the capital city of Hebei province in northeast China in July 2021. These greenhouses were built 3–8 years prior to sample collections in 2021. Before these greenhouses were built, the land was farmland used for growing corn (during the summer) and wheat (during the winter). In these greenhouses, tomatoes, cucumbers, and eggplants were commonly planted and rotated among them every 2–3 years. At the time of our soil sampling, these greenhouses were used for producing batatas, tomatoes, cucumbers, and eggplants (Table S1). The detailed information on collection sites is shown in Table S1. Within each greenhouse, 100 soil samples separated at least 100 cm from each other were collected and put into zip-lock plastic bags. Fungal strains resembling *A. alternata* were isolated and purified from each soil sample following the method described previously by Yang et al. (21). Briefly, for each soil sample, 1–2 g of soil was suspended in 1.5 mL sterile water and well mixed under 180 rpm for 30 min. Subsequently, 1 mL of this suspension was transferred to a petri dish and mixed with 10 mL of 55°C modified potato carrot agar (PCA; 10 g carrot, 10 g potato, and 20 g agar per L medium, modified with 30 mL 2% MnCl_2_·4H_2_O and 50 mg/L chloramphenicol). Solidified medium with PCA and soil suspension was incubated in the dark at 28°C for 5 days. Based on species-specific microscopic morphological features, single spores of typical *Alternaria* colonies were picked using the tip of a very thin needle and transferred to new PCA plates. To minimize isolating strains of the same genotype and phenotype from the same soil sample, a maximum of one isolate was obtained from each agar plate or each soil sample.

Genomic DNA was extracted from the mycelia of each isolate using the cetyltrimethylammonium bromide procedure (34). All isolates were checked morphologically under a light microscope and molecularly by PCR amplification and sequencing of the internal transcribed spacer (ITS) regions of the ribosomal RNA gene cluster to confirm their identity as belonging to *A. alternata*. In addition, following the method previously described by Woudenberg et al. (35–37), we randomly took 13 putative *A. alternata* isolates from across the seven greenhouses and obtained their sequences at the following six loci for species confirmation: glyceraldehyde-3-phosphate dehydrogenase (*gapdh*), RNA polymerase second largest subunit (*rpb2*), translation elongation factor 1-alpha (*tef1*), *Alternaria* major allergen gene (*Alta1*), endopolygalacturonase (*endoPG*), and an anonymous gene region (*OPA10-2*).

### Triazole susceptibility testing

To investigate triazole susceptibilities of *A. alternata* isolates, we selected four triazoles, two first-line clinical triazole drugs, itraconazole (ITR) and voriconazole (VOR), and two agricultural triazole fungicides, tebuconazole (TEB) and difenoconazole (DIF). Fungicides TEB and DIF are widely used in agriculture for controlling plant diseases caused by *A. alternata* and other fungal pathogens. Medical triazole ITR (98% purity) was purchased from Shanghai Macklin Biochemical Technology Co., Ltd. (Shanghai, China), while VOR (98% purity), TEB (99% purity), and DIF (97%) were purchased from Shanghai Yuanye Bio-Technology Co., Ltd. (Shanghai, China). Following the method described in the CLSI M38-A2 (38), susceptibilities of each *A. alternata* isolate to the four triazoles were determined. First, stock solutions of 1.6 × 10^3^ µg/mL of all four triazoles were prepared by dissolving the triazoles in dimethyl sulfoxide, respectively. Second, wells of flat-bottomed microtiter plates were filled with 100 µL of RPMI 1640 medium amended with a series of twofold dilutions of drugs, with the concentrations of all four triazoles ranging from 0.0156 to 32.00 μg/mL. Prior to amending with the triazoles, the RPMI 1640 medium was adjusted to pH 7 using 0.165 M 3-N-morpholinepropanesulfonic acid. Then, 100 µL conidial suspension of each strain was added into each well to make the final triazole concentration range from 0.0078 to 16.00 mg/mL in twofold dilution series in the 96-well microtiter plate. After 72-h incubation at 30°C, the MIC value of each isolate was determined based on the lack of mycelial growth on conidial spores based on microscopic observations. The concentration of each triazole agent showing no mycelial growth represented the MIC value for the specific strain to the tested triazole agent (21).

### Sequence analysis of the triazole target gene *cyp51*

Potential genetic alternations in the *A. alternata cyp51* gene that encodes the target of triazoles were investigated based on the complete and partial sequences of the gene. Specifically, the gene from each isolate was amplified using the following primers: Altcyp51-F (5′- ATTGGATACCCTGGTCCATGC-3′) and Altcyp51-R (5′-TTAAGACCCGAAATGCGTCG-3′) (24). PCR amplification conditions comprised 95°C for 4 min, 32 cycles of 30 s at 95°C, 30 s at 62°C, and 2 min at 72°C, followed by a final extension step of 5 min at 72°C. PCR products were separated in 1.0% agarose gels in 1× Tris-acetate-EDTA buffer (40 mM Tris-acetate and 1 mM EDTA, pH 8.0) and purified using a gel extraction kit (TransGen Biotech). The PCR fragment was sequenced with both the forward and reverse primers Altcyp51-F and Altcyp51-R, respectively (Tsingke Biotechnology Co., Ltd., Kunming, China). DNA sequences were analyzed with MEGA 11. Mutations in the *cyp51* gene were identified by comparing our sequences (GenBank accession numbers PP731575-PP731778) with the reference sequence of a triazole-susceptible *A. alternata* strain in GenBank with the accession number MN542658 (24). The potential cross-resistance among the four triazoles was investigated by comparing each strain’s MIC values across the four triazoles and by using the Pearson correlation analysis based on their MIC values at the population level. Pearson correlation analyses were carried out using IBM SPSS statistical software version 25.0.

### Microsatellite genotype analysis

All 233 *A*. *alternata* isolates were genotyped at 10 microsatellite markers (c10062, c9473, c10524, c9860, c3806, c10756, AEM6 DQ272485, AEM9 DQ272486, PAS2, and PAS6). These markers were previously shown to have high amplification success and abundant polymorphisms in *A. alternata* populations from Yunnan greenhouses (21).

For each isolate, its allele at each locus was assigned based on the size of the PCR amplicon generated by each pair of SSR primers using the ABI 3730 DNA sequencer (Applied Biosystems). PCR amplification with an identical size generated by the same pair of primers was considered as the same allele. Locus-based diversity indices across the entire population including major allele frequency (MAF), number of alleles, and polymorphic information content (PIC) were computed using PowerMarker v3.25 software (39). Genetic diversity within individual subpopulations was evaluated by gene diversity (40), allele number, and standardized Shannon index (41) using the program Popgene v.1.32. Clone correction was carried out based on their multilocus genotypes, where one representative isolate of each genotype in each greenhouse was kept and used for further analysis. The potential genetic differentiation among subpopulations was evaluated using a hierarchical analysis of molecular variance (AMOVA) performed in GenAlEx v 6.501 (42). Total genetic variation was calculated first among subpopulations within Shijiazhuang (SJZ 1–SJZ 7). In addition, we analyzed the Kunming (242 isolates in nine greenhouses, 192 isolates remained after clone correction) and Shijiazhuang greenhouse populations together at three hierarchical levels: among regions (Kunming and Shijiazhuang), among greenhouse subpopulations within regions (SJZ.1–SJZ.7 and YN.1–YN.9), and among strains within individual greenhouse subpopulations. To further explore the genetic structure of *A. alternata* populations, we used the program STRUCTURE version 2.3.3 to estimate the optimal number (*K*) of genetic clusters in the Shijiazhuang population and in the combined total samples from both Kunming and Shijiazhuang. The relationships among strains were estimated using Ward’s method and shown in a phylogeny (43). K-means hierarchical clustering and discriminatory analysis of principal components (DAPC) were performed using R program, version 4.1.3. Evidence for recombination within individual genetic populations was assessed using phylogenetic incompatibility and linkage equilibrium tests, following the protocols described in an earlier study (21).

## RESULTS

### Isolates and identification

A total of 233 single spore isolates of *A. alternata* were obtained from the soil samples of seven greenhouses collected from Shijiazhuang, northeast China. Among them, 41, 13, 29, 38, 40, 36, and 36 isolates were obtained from the seven greenhouses (SJZ.1–SJZ.7), respectively. To further identify the isolates at the species level, Bayesian analyses based on ITS sequences of all isolates and sequences at six loci (*gapdh*, *rpb2*, *tef1*, Alt a 1, endoPG, and OPA10-2) for 13 randomly selected isolates, together with DNA sequences of representative species within the *Alternaria* genus, were conducted. For these 13 isolates in our study, the length of the combined sequences of the seven genes was 3,505 bp, with the lengths of individual genes ranging from 241 bp (*tef1*) to 753 bp (*rpb2*). Isolates in this study were clustered in the clade containing known reference isolates of *A. alternata* and morphospecies that have since been synonymized as *A. alternata* (Fig. S1). Interestingly, our phylogenetic analyses identified two distinct lineages, which we call clade A and clade B, within *A. alternata* with 100% bootstrap support. In addition, clade A contained representative strains in this study that could be further divided into two subclusters (Fig. S1).

### Genotypic diversity and population structure of *A. alternata* isolates from greenhouses in China

The 10 STR primer pairs were originally developed for *Alternaria tenuissima* (44), *A. alternata* (45), and *Alternaria solani* (46). Here, these 10 loci had polymorphic information content ranging from 0.09 (AEM9 DQ272486) to 0.83 (PAS2) in the Shijiazhuang greenhouse populations of *A. alternata* (Table 1). A previous study classified the polymorphism level of STR markers based on PIC values into low (PIC < 0.25), medium (0.25 < PIC < 0.5), and high (PIC > 0.5) (47). Based on this criterion, among the 10 STR loci [which all showed high-level polymorphism in the Yunnan greenhouse populations (Table 1)], two (AEM9 DQ272486 and PAS6) had low PIC, five (c10062, c9473, c9860, c3806, and c10756) had moderate PIC, and the remaining three (c10524, AEM6 DQ272485, and PAS2) had high PIC for the Shijiazhuang regional population (Table 1) For the combined populations from Kunming and Shijiazhuang provinces, two STR loci (AEM9 DQ272486 and PAS6) had moderate PICs, while the remaining eight showed high PICs. The highest discriminatory power value for a single locus was observed with PAS2, with a gene diversity value of 0.87 and a total of 19 observed alleles (Table 1). Diversity of c10062 (*P* = 0.043), c9473 (*P* = 0.042), c10756 (*P* = 0.0063), AEM9 DQ272486 (*P* < 0.0001), and PAS6 (*P* = 0.0002) in Yunnan populations was significantly higher than Shijiazhuang populations (Fig. S2).

**TABLE 1:** Allelic richness and diversity across 10 microsatellite loci in the Kunming and Shijiazhuang greenhouse populations of *A. alternata*

Locus no.	Number of alleles			MAF^*b*^			PIC^*c*^			Gene diversity		
	KM^*a*^	SJZ^*a*^	Total	KM	SJZ	Total	KM	SJZ	Total	KM	SJZ	Total
c10062	10 (7)	3	10 (7)	0.378	0.730	0.648	0.59	0.40	0.52	0.63	0.43	0.55
c9473	7 (3)	4	7 (3)	0.370	0.639	0.600	0.59	0.40	0.53	0.63	0.49	0.57
c10524	17 (7)	11 (1)	18 (8)	0.144	0.305	0.276	0.84	0.77	0.82	0.85	0.79	0.84
c9860	6 (3)	3	6 (3)	0.353	0.627	0.573	0.6	0.48	0.55	0.64	0.54	0.60
c3806	15 (8)	17 (10)	25 (18)	0.398	0.665	0.629	0.57	0.47	0.55	0.60	0.51	0.57
c10756	8(4)	7(3)	11 (7)	0.261	0.730	0.560	0.7	0.43	0.61	0.74	0.45	0.64
AEM6DQ272485	14 (7)	8(1)	15 (8)	0.220	0.438	0.331	0.75	0.64	0.73	0.78	0.69	0.77
AEM9DQ272486	8 (4)	5 (1)	9 (5)	0.419	0.953	0.749	0.51	0.09	0.38	0.58	0.09	0.41
PAS2	12 (1)	18 (7)	19 (8)	0.149	0.249	0.234	0.83	0.83	0.85	0.85	0.85	0.87
PAS6	6 (3)	4 (1)	7 (4)	0.437	0.931	0.762	0.5	0.13	0.37	0.56	0.13	0.40
Total	103 (47)	80 (24)	127 (71)	0.313	0.627	0.536	0.648	0.46	0.59	0.69	0.50	0.62

^*a*^ KM, Kunming; SJZ, Shijiazhuang; values in parenthesis are the number of private alleles found only in one geographic region.

^*b*^ MAF, Major allele frequency.

^*c*^ PIC, polymorphism information content.

Given the observed allele numbers and assuming recombination, the total possible number of multilocus genotypes at the 10 microsatellite loci in the Shijiazhuang population of *A. alternata* is 3 × 4 × 11 × 3 × 17 × 7 × 8 × 5 × 18 × 4 = 135,717,120. For the Kunming greenhouse population, the total number of possible genotypes at the same 10 microsatellite loci is 10 × 7 × 17 × 6 × 15 × 8 × 14 × 8 × 12 × 6 = 6,909,235,200. The high number of possible genotypes means that the probability of having strains with identical genotypes at the 10 loci by chance and not due to clonal reproduction is extremely low, at 1/135,717,120 and 1/6,909,235,200, respectively, for the Shijiazhuang and Kunming greenhouse populations. In the remaining analyses, when appreciated, clone-corrected samples were also analyzed, along with the total samples.

For the 233 *A*. *alternata* isolates from seven greenhouses in Shijiazhuang, a total of 80 alleles were identified at the 10 microsatellite loci with 27 of the 80 alleles found in only one of the seven greenhouses (Table 2). For the 27 private alleles, five, two, two, seven, four, four, and three alleles were observed in greenhouses SJZZ.1–SJZ.7, respectively (Table S2). The total number of alleles per locus in the Shijiazhuang population ranged from 3 (c9860) to 18 (PAS2), with an average allele number of 8 among the 10 SSR loci. The average Nei’s gene diversity for all SSR markers was 0.63, varying from 0.09 (AEM9 DQ272486) to 0.85 (PAS2), allelic evenness ranged from 0.35 (AEM9 DQ272486) to 0.79 (c9473) (Table 2). The gene diversity of seven greenhouse populations ranged from 0.40 (SJZ.2) to 0.50 (SJZ.7), with an average gene diversity of 0.46 (Table 2). The 10 SSR markers differed in the number of private alleles among the seven greenhouses, with the largest number found at locus c3806 with 12 private alleles, while no private allele was found at loci c10062, c9860, and PAS6 (Table 2).

**TABLE 2:** Allele distributions and genetic diversity within and among the seven greenhouse populations of *Alternaria alternata* for each of the 10 STR loci

Population	Number of isolates (genotypes)	Gene diversity	Number of alleles at each locus (number of private alleles in parenthesis)										
			c10062	c9473	c10524	c9860	c3806	c10756	AEM6 DQ272485	AEM9 DQ272486	PAS2	PAS6	Total
SJZ.1	41 (35)	0.47	3	4 (1)	8	3	5 (1)	5	6 (1)	2	11 (2)	1	48 (5)
SJZ.2	13 (12)	0.40	2	2	5	3	2	3	4	2 (1)	6 (1)	2	31 (2)
SJZ.3	29 (28)	0.49	3	3	7	3	5 (2)	5	4	2	7	2	41 (2)
SJZ.4	38 (34)	0.48	3	3	11 (2）	3	6 (3)	4	6 (2)	1	10	3	50 (7)
SJZ.5	40 (38)	0.47	2	2	7	3	6 (2)	7 (1)	4	2	12 (1)	2	47 (4)
SJZ.6	36 (31)	0.43	3	2	6	3	6 (3)	4	4	1	11 (1)	2	42 (4)
SJZ.7	36 (23)	0.50	3	3	6	3	3 (1)	6	5	3 (1)	7 (1)	3	42 (3)
Total	233 (171)	0.46	3	4 (1)	11 (2）	3	17 (12)	7 (1)	8 (3)	5 (2)	18 (6)	4	80 (27)

Similar to the total Shijiazhuang population, the number of possible multilocus genotypes (MLGs) within each greenhouse is also very high, with the smallest in SJZ.2 being 34,560 and the largest in SJZ.4 being 11,547,360. However, for the Shijiazhuang greenhouse populations, of the 233 isolates, we found 171 MLGs across the 10 SSRs. Among these, 30 MLGs were shared between at least two greenhouses, and each of the remaining 141 MLGs was found in only one greenhouse. Together, the results suggest evidence for clonal expansion of *A. alternata* genotypes within and between greenhouses in Shijiazhuang.

Across the seven greenhouses, 74% of the MLGs were found only in one greenhouse. The highest frequency of private MLGs (94%, 32/34) was in SJZ.4 and the lowest was in SJZ.5 (58%, 22/38) (Table S2). The chi-square goodness-of-fit test (χ^2^ test) was used to evaluate the significance of the difference in the number of private MLGs. The results showed that there was significant difference between SJZ.1 and SJZ.4 (*P* = 0.013), SJZ.3 and SJZ.4 (*P* = 0.003), SJZ.4 and SJZ.5 (*P* = 0), SJZ.4 and SJZ.6 (*P* = 0.013), and SJZ.5 and SJZ.7 (*P* = 0.018). Similar patterns were observed in Kunming greenhouse populations. Specifically, across the nine greenhouses in Kunming, 76% of the total MLGs were found only in one greenhouse with the highest found in YN.7 (96%, 25/26) and the fewest in YN.3 (53%, 8/15). In addition, significant differences in the prevalence of private MLGs were observed between YN.7 and several other greenhouse populations including YN.1 (*P* = 0.004), YN.2 (*P* = 0.029), YN.3 (*P* = 0.001), and YN.5 (*P* = 0.037). Mantel test showed no significant association between the distribution of private genotypes within greenhouses and the geographical distances among them in both the Kunming and Shijiazhuang populations (Fig. S3 and S4).

For the combined total of 475 isolates from 16 greenhouses (242 isolates from nine greenhouses in Kunming + 233 isolates from seven greenhouses in Shijiazhuang), a total of 127 alleles were found at the 10 SSR loci (Table 1). Among them, 56 alleles were shared between those two geographical populations. Of the remaining 71 alleles, 47 and 24 alleles were unique to Kunming and Shijiazhuang populations, respectively (Table 1). A total of 336 MLGs were detected among the 475 isolates based on the 10 SSR loci. Among these 336 MLGs, two were shared between these two geographical regions, consistent with long-distance dispersals.

**Fig 1:**
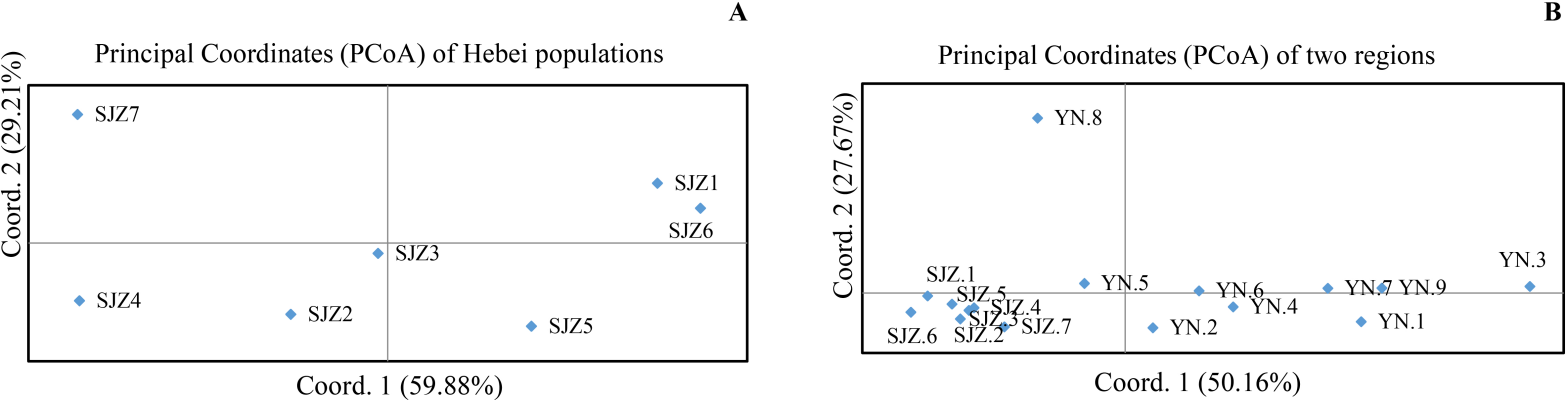
Result of principal coordinate analyses. (**A**) Principal coordinate analysis based on pairwise population genetic distances of seven populations (greenhouses) in Shijiazhuang, Hebei. (**B**) Principal coordinate analysis based on pairwise population genetic distances of 16 greenhouses from Kunming, Yunnan and Shijiazhuang, Hebei.

AMOVA of all isolates from seven greenhouses Shijiazhuang showed that 97% of the total genetic variation was attributable to variations within individual greenhouses, and low (3%) but statistically significant variation was due to separation among the seven greenhouses (PhiPT = 0.027, *P* = 0.001) (Table S3). After clone correction, low (~1% of the total) but statistically significant genetic variation was attributed to variations among greenhouses (PhiPT = 0.012, *P* = 0.026) (Table S4). The extent of genetic differentiation between pairs of greenhouse populations was further investigated. The result showed differentiation between several greenhouse pairs. The biggest differentiation was observed between SJZ.1 and SJZ.4 (Fst = 0.043, *P =* 0.002), followed by that between SJZ. 4 and SJZ.6 (Fst = 0.040, *P =* 0.001) and SJZ.1 and SJZ.2 (Fst = 0.024, *P =* 0.102) (Table S5). However, the Mantel test showed no significant correlation between geographical distance and genetic distance among the seven greenhouse populations of *A. alternata* in Shijiazhuang (Fig. S5). Principal coordinate analysis (PCoA) based on pairwise genetic distances between greenhouse populations revealed that the first and second principal coordinate axes explained 75.81% and 15.38% of the total variation, respectively (Fig. 1A). Finally, allelic association analyses revealed evidence for non-random recombination in both the total population (PrC = 0.11; rBarD = 0.023, *P*＜0.001) and in most individual greenhouse populations such as SJZ.1 (PrC = 0.38; rBarD = 0.029, *P* = 0.021), SJZ.4 (PrC = 0.40; rBarD = 0.055, *P* = 0.001), and SJZ.6 (PrC = 0.42; rBarD = 0.96, *P* = 0.012).

AMOVA performed on all isolates from both northeast and southwest China showed that 86% of the total genetic variation was found within individual greenhouses, while 7% and 7% were, respectively, found among greenhouse populations from within each region and between the two distant regions (PhiPT = 0.138, *P* = 0.001) (Table S6). Among the 120 [(16 × 15)/2 = 120] greenhouse population pairs, 100 pairs showed statistically significant differentiation (*P* ＜0.05). Among them, the subpopulations with the highest degree of genetic differentiation were observed between YN.3 and SJZ.6 (PhiPT = 0.236, *P* = 0.001). Interestingly, the differentiation between YN.8 and other Kunming subpopulations was as large as the observed differentiation between YN.8 and all seven subpopulations from Shijiazhuang (Table S5). Overall, there was a weak but statistically significant correlation between greenhouse subpopulation genetic distances and geographic distances (*r* = 0.0042, *P* = 0.01) (Table S6). Consistently, the dendrogram generated using the unweighted pair group method with arithmetic mean weakly grouped the nine Kunming subpopulations into three major clusters (Fig. S7), with YN.8 located in distinct genetic cluster.

**Fig 2:**
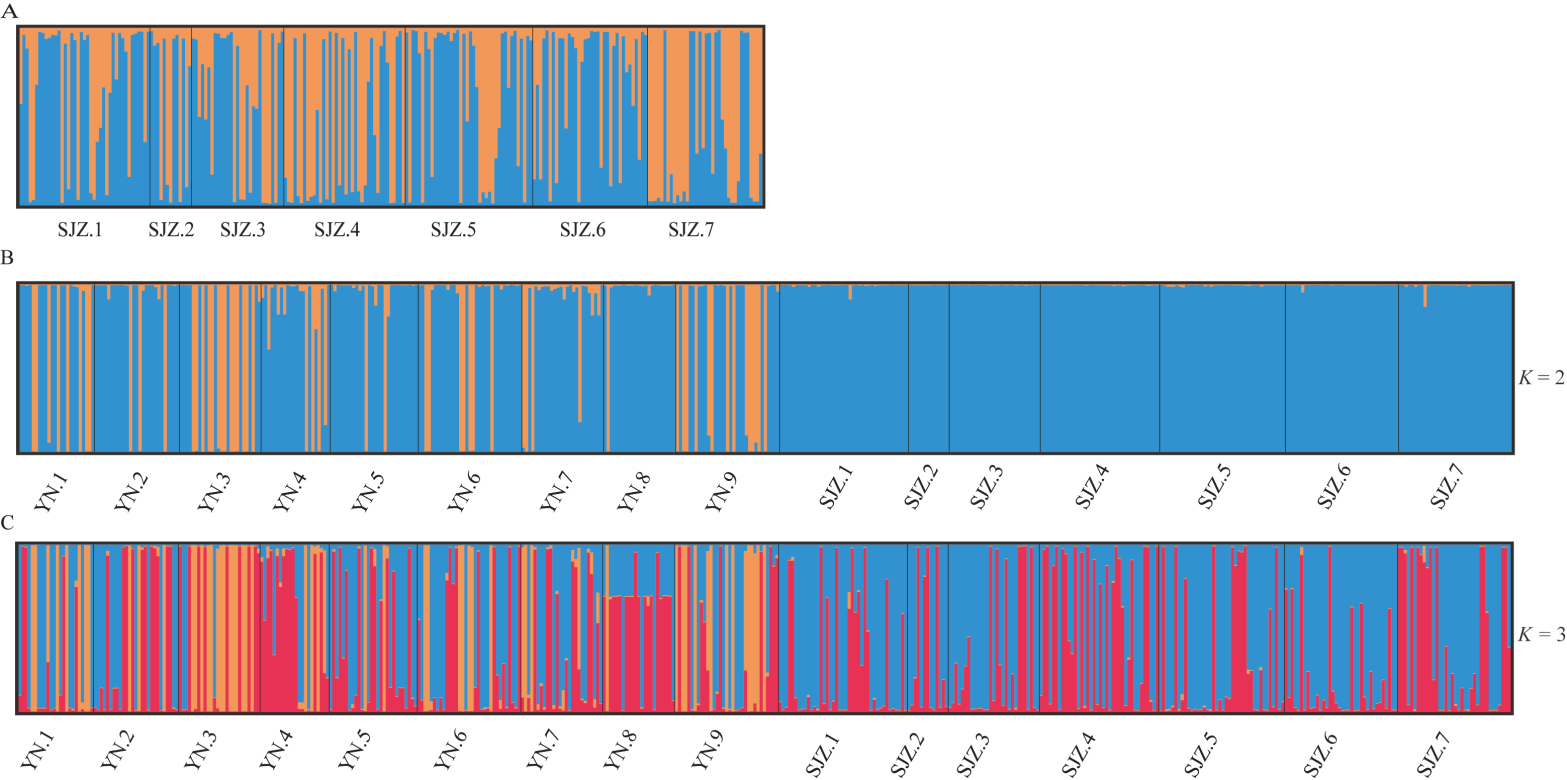
Population genetic clusters inferred with the STRUCTURE software. (**A**) Strains from Shijiazhuang. (**B**) Strains from Kunming and Shijiazhuang for *K* = 2. (C) Strains from Kunming and Shijiazhuang for *K* = 3.

Based on the PCoA, all seven subpopulations from Shijiazhuang clustered within the two right quadrants of the first PCoA axis (Fig. 1B), and the nine Kunming greenhouse populations showed broader spread across the first component space (explaining 65.42% of the variation), having no overlap with any of the Shijiazhuang subpopulations. Subpopulation YN.8 in Kunming and other 15 subpopulations fell into two different clusters in the second PCoA axis (Fig. 2B; Table S3). For the total population with combined isolates from both geographic regions, the hypotheses of strict clonality and random recombination were both rejected (rBarD = 0.137, *P*＜0.001), consistent with non-random recombination in this sample of *A. alternata*.

### Evidence for distinct genetic populations of *A. alternata* in greenhouse soils

To further infer the potential population structure of *A. alternata* populations, we estimated the optimal number of genetic clusters in the Shijiazhuang greenhouses and the total sample based on the Bayesian model implemented in STRUCTURE. Output of STRUCTURE Harvester revealed that the delta *K* (Δ*K*) value reached a sharp peak at *K =* 2, confirming that the isolates from Shijiazhuang are optimally clustered into two subpopulations (Fig. 2A). Similarly, STRUCTURE analyses of population from the combined southwest and northeast China also identified two distinct genetic clusters (Fig. 2B). At *K* = 2, the Kunming populations had a mixture of two elements (represented by blue and orange), while the Shijiazhuang population mostly belonged to one element (represented by blue) (Fig. 2B). At *K* = 3, two genetic components (represented by light blue and red) were widespread in Kunming and Shijiazhuang populations, while the third component (light orange) was mainly distributed in Kunming greenhouses (Fig. 2C).

**Fig 3:**
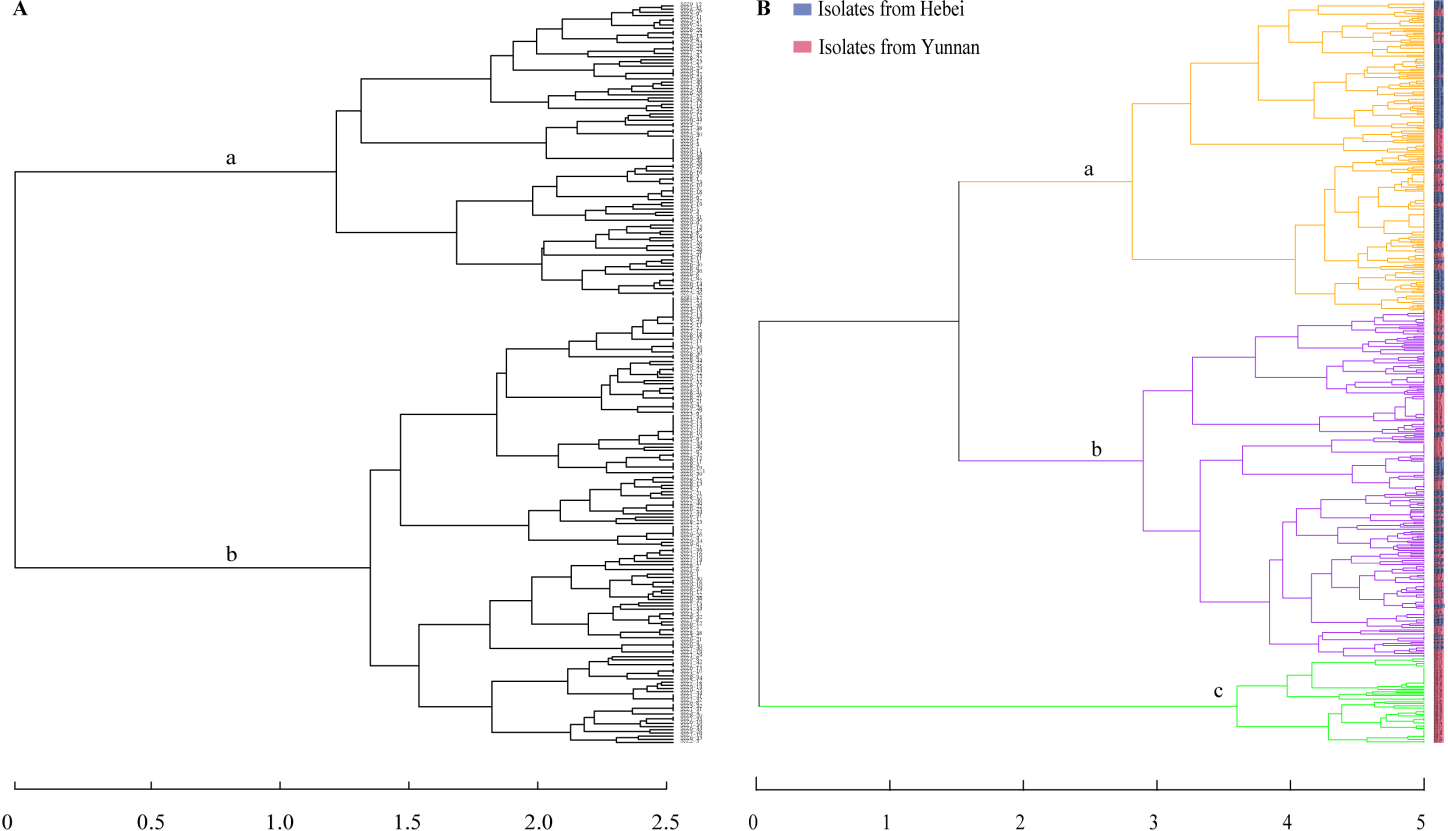
Dendrogram representing the hierarchical clustering of isolates by Ward’s method. (**A**) Strains from Shijiazhuang. (**B**) Strains from Kunming and Shijiazhuang. Three clades (a–c) were identified in Fig. 4B as labeled.

**Fig 4:**
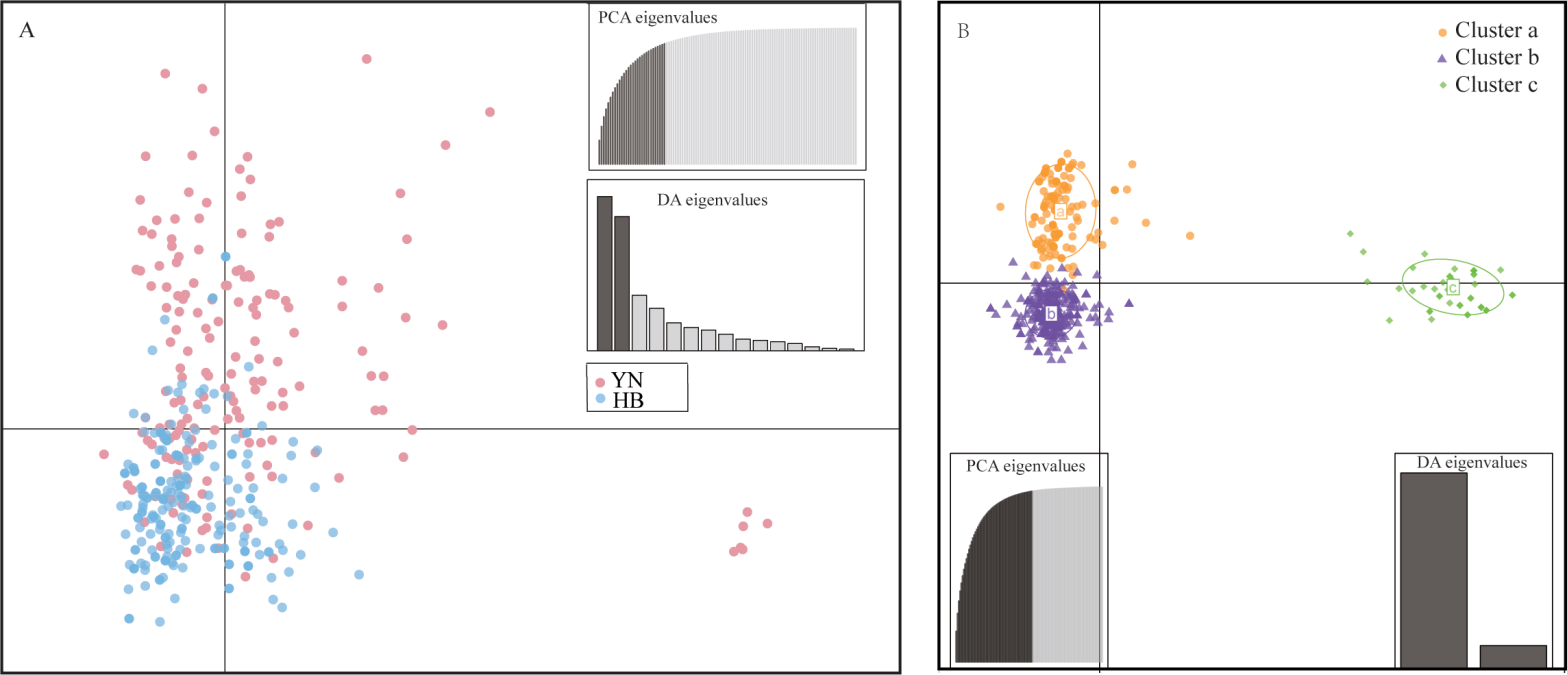
Discriminant analysis of principal components among strains of *A. alternata* from Kunming, Yunnan, and Shijiazhuang, Hebei provinces. (**A**) DAPC analysis carried out in “poppr” package and 15 discriminant functions saved. (**B**) DAPC analysis carried out in “adegenet” package, and three clusters were achieved by “find clusters” function, which correspond to clades indicated in Fig. 3B.

Hierarchical clustering of the Shijiazhuang isolates based on Bruvo’s distances by Ward’s method further identified two broadly divergent clades, with 92 (39.48%) isolates being assigned to clade a and 141 (60.52%) being assigned to clade b (Fig. 3A). However, when isolates from Kunming were added, three clusters were recognized, with 216 (45.47%) being assigned to clade a, 203 (42.74%) isolates to clade b, and 56 (11.79%) isolates (all from Kunming) assigned to clade c (Fig. 3B). Of these three clades, clades a and b corresponded to the two sister clades in lineage A, and clade c corresponded to lineage B as shown in the phylogeny based on concatenated sequence of seven genes (Fig. S1). DAPC indicated a similar pattern of differentiation, consistent with the three clusters identified based on hierarchical clustering and concatenated phylogenetic analyses (Fig. 4). The presence of genetically differentiated clades within greenhouse populations in both Kunming and Shijiazhuang was supported by individualized analyses between pairs of clades that showed moderate to high differentiation, i.e., moderate between clades a and b (PhiPT = 0.144, *P* = 0.001), and high-level differentiations between clades a and c (PhiPT = 0.4336, *P* = 0.001) and between clades b and c (0.510, *P* = 0.001). The index of association (Ia) was used to quantify the extent of linkage disequilibrium between SSR loci within each clade. The result showed non-random associations among alleles in clade a (Ia = 0.319, *P* = 0.001), clade b (Ia = 0.102, *P* = 0.03), and clade c (Ia = 1.421, *P* = 0.001). However, unambiguous evidence for recombination in clade a (PrC = 0; rBarD = 0.043, *P* = 0.012), clade b (PrC = 0.022; rBarD = 0.0001, *P* = 0.423), and clade c (PrC = 356; rBarD = 138, *P* = 0.001) was observed. Together, the results indicate significant genetic differentiation among the three clades and evidence for (non-random) recombination within each of the three clades.

### Susceptibility of *A. alternata* isolates to triazoles

Among the 233 isolates, 211 produced sufficient conidia for drug susceptibility testing, while the remaining 22 produced very limited conidia, and we were unable to obtain their antifungal susceptibility information. All seven greenhouses were represented by the 211 isolates. The susceptibilities for each of the 211 isolates to two medical triazole drugs (VOR and ITR) and two agricultural triazole fungicides (DIF and TEB) were analyzed using a twofold serial dilution of drug concentrations from 16.00 to 0.0078 μg/mL in a microtiter plate format in this study. The results showed that the MIC values of the 211 isolates to ITR and DIF ranged from 0.0078 to ≥16.00 µg/mL, and the ranges of MICs to TEB and VOR were 0.125 –≥16.00 µg/mL (Table 3). The MIC_50_ of 211 isolates to VOR, ITR, DIF, and TEB were 4, 1, 1, and 8 µg/mL, respectively. Relatively few isolates, 3.32% (7/211), 1.42% (3/211), and 14.2% (30/211), had MICs of ≤0.125 µg/mL to ITR, TEB, and DIF, respectively. Most isolates, 78.7% (166/211), 82.5% (175/211), and 76.3% (161/211), had MIC values of 0.125–4 µg/mL to ITR, VOR, and DIF, respectively (Table 3). In contrast, 78.2% (165/211) of the isolates had MIC of ≥8 µg/mL to TEB, while 9.5% (20/211) of the isolates had MIC of ≥8 µg/mL for DIF.

**TABLE 3:** Distributions of triazole susceptibilities among *Alternaria alternata* samples from seven greenhouses in Shijiazhuang, Hebei, China

Pop.	Proportion of strains (no. of isolates within MIC value range/total no. of isolates) within each MIC range of the following four triazoles:										
	ITR			VOR		TEB			DIF		
	<0.125 µg/mL	0.125–4 μg/mL	≥8 µg/mL	0.125–4 μg/mL	≥8 µg/mL	<0.125 µg/mL	0.125–4 μg/mL	≥8 µg/mL	<0.125 µg/mL	0.125–4 μg/mL	≥8 µg/mL
SJZ.1	17.95 (7/39)	71.79 (28/39)	10.26 (4/39)	89.74 (35/39)	10.26 (4/39)	5.13 (2/39)	58.97 (23/39)	35.9 (14/39)	51.28 (20/39)	41.03 (16/39)	7.69 (3/39)
SJZ.2	0	63.64 (7/11)	36.36 (4/11)	81.82 (9/11)	18.18 (2/11)	0	27.27 (3/11)	72.73 (8/11)	36.36 (4/11)	63.64 (7/11)	0
SJZ.3	0	92.31 (24/26)	7.69 (2/26)	76.92 (20/26)	23.08 (6/26)	0	7.69 (2/26)	92.31 (24/26)	0	84.62 (22/26)	15.38 (4/26)
SJZ.4	0	68.57 (24/35)	31.43 (11/35)	82.86 (29/35)	17.14 (6/35)	0	17.14 (6/35)	82.86 (29/35)	5.71 (2/35)	88.57 (31/35)	5.71 (2/35)
SJZ.5	0	87.18 (34/39)	12.82 (5/39)	82.05 (32/39)	17.95 (7/39)	0	5.13 (2/39)	94.87 (37/39)	0	92.31 (36/39)	7.69 (3/39)
SJZ.6	0	74.19 (23/31)	25.81 (8/31)	90.32 (28/31)	9.68 (3/31)	0	16.13 (5/31)	83.87 (26/31)	6.45 (2/31)	83.87 (26/31)	9.68 (3/31)
SJZ.7	0	86.67 (26/30)	13.33 (4/30)	73.33 (22/30)	26.67 (8/30)	3.33 (1/30)	6.67 (2/30)	90 (27/30)	6.67 (2/30)	76.67 (23/30)	16.67 (5/30)
Total	3.32 (7/211)	78.67 (166/211)	18.01 (38/211)	82.94 (175/211)	17.06 (36/211)	1.42 (3/211)	20.38 (43/211)	78.2 (165/211)	14.22 (30/211)	76.3 (161/211)	9.48 (20/211)

The seven greenhouse populations in Shijiazhuang often showed highly variable susceptibility patterns to the same triazoles. For example, 7.7% (2/26) isolates in SJZ.3 had an MIC value of ≥8 µg/mL to ITR, but 36.4% (4/11) and 30% (11/35) in SJZ.2 and SJZ.4 had the same MIC value to ITR. The highest and lowest frequencies of MIC ≥ 8 µg/mL to VOR were observed in SJZ.7 (26.7%, 8/30) and SJZ.6 (9.7%, 3/31). In SJZ.7, we also observed the highest frequency of MIC value of ≥8 µg/mL to TEB (90%, 27/30) and DIF (16.7%, 5/30). However, isolates from the same greenhouse can also have variable MIC distribution patterns among the tested triazoles. For example, 90% (27/30) isolates in SJZ.7 had MIC values of ≥8 µg/mL to TEB, while less than 30% had the same MIC value to ITR, VOR, and DIF.

The χ^2^ test was used to examine the differences between greenhouses in proportion of resistance to four tested triazole fungicides. The result showed no significant differences among the seven greenhouses in their frequency of resistance to VOR and DIF. However, significant differences in the frequency of resistance to TEB and ITR were found between several greenhouses. Specifically, greenhouse pairs that showed significant differences in ITR susceptibilities include SJZ.1 and SJZ.2 (*P* = 0.037), SJZ.1 and SJZ.4 (*P* = 0.024), SJZ.2 and SJZ.3 (*P* = 0.031), and SJZ.3 and SJZ.4 (*P* = 0.025). The greenhouse pairs showing significant differences to TEB were observed between SJZ.1 and other greenhouses (*P* < 0.05). However, in Yunnan populations, there were more greenhouse pairs that showed significant differences, even to DIF and VOR (21).

Pearson correlation analysis based on their MIC values to different triazoles was used to investigate the potential relationship among susceptibilities to the four triazoles at the population level. Our results showed statistically significant positive correlations between all paired comparisons, with coefficients of correlation of 0.297 (*P =* 0), 0.399 (*P =* 0), 0.366 (*P =* 0), 0.503 (*P =* 0), 0.418 (*P =* 0), and 0.382 (*P =* 0) between ITR and VOR, ITR and TEB, ITR and DIF, TEB and VOR, DIF and VOR, and DIF and TEB, respectively (Table S7). Together, this result indicates evidence for at least some shared mechanisms of resistance to the four triazoles at the population level for the greenhouse populations of *A. alternata* in Shijiazhuang.

### *Cyp51* nucleotide sequence variation

Using primer pair Altcyp51-F/Altcyp51-R, a fragment of 1,673 bp was successfully amplified and sequenced in 204 isolates, including two 49-bp introns and amino-acid-coding regions. Our sequence comparisons revealed a total of 40 single nucleotide polymorphisms (SNPs), and these SNPs clustered the 204 isolates into 16 sequence types at the *cyp51* locus. Among the 40 SNPs, two and eight SNPs were located within the first and second introns, respectively, and the remaining 30 SNPs were in exons with 9 being nonsynonymous and 21 synonymous. For the 21 synonymous substitutions, nine (9/21, 43%) were observed in only one strain each and were thus found in only one greenhouse each; nine other synonymous substitutions (9/21, 43%, including 117C→T, 370C→T, 466C→T, 469C→T, 572A→T, 575C→T, 671A→G, 1304C→G, and 1364C→T) were each observed in all seven greenhouse populations, and the remaining three mutations (334G→A, 881C→T, and 995A→C) were shared by two to six greenhouses (3/21, 14%). For the 10 SNPs observed in introns, mutation 514C→T was the most common type with overall frequencies of 46% (94/204); two mutations (507 C→T and 519 A→G) were observed in only one strain each, and a combination of mutations [at 288A→G, 289G→A, 526 G→A, 533G→A, and a triplet code deletion (504, 505, and 506 bp)] were observed in every greenhouse population.

The nine nonsynonymous SNPs resulted in seven amino acid substitutions: N188K, V192I, S237A, A307G, L323W, G448S, and G462S. Among these mutations, one isolate had a combination of five substitutions, N188K, V192I, S237A, A307G, and G448S. Another isolate had a combination of two mutations L323W and G462S. Amino acid substitution G462S was widespread in every greenhouse population with an overall frequency of 18% (36 of 204 strains with successfully amplified *cyp51* gene).

The chi-square goodness-of-fit test found that amino acid substitutions of G462S were significantly associated with the frequencies of *A. alternata* isolates distributed in MIC ≤ 4 µg/mL and MIC ≥ 8 µg/mL to VOR (*P* = 0) and TEB (*P* = 0.046), respectively. In 150 VOR-sensitive isolates, five isolates carried the G462S mutation, while among 31 VOR-resistant isolates, 22 had the G462S mutation. For TEB, 5% (2/40) and 17.7% (25/141) isolates contained the G462S mutation in sensitive and tolerant isolates, respectively. However, for the same MIC ranges to ITR and DIF, no statistically significant association was detected with G462S.

## DISCUSSION

### Isolation by greenhouses contributes significantly to genetic differentiation among *A. alternata* populations

In this study, we obtained isolates of *A. alternata* from greenhouse soils and used a panel of 10 STR markers to identify the genotypes of these isolates and to analyze the genetic diversity within and relationship among populations of this species. Our results suggest that greenhouses can significantly impact the structure and evolution of soil populations of *A. alternata*. First, in both the Kunming greenhouses reported in our previous study and the Shijiazhuang greenhouses reported here in this study, a large number of alleles and genotypes were found in each greenhouse. Indeed, in the hierarchical analyses of molecular variance, most of the genetic variations were found within individual greenhouses. Second, all individual greenhouses contained private alleles in at least 1 of the 10 loci, some of them at relatively high frequency, consistent with independent evolution among them. Third, in the total population containing isolates from both southwest and northeast China, despite the considerable distance between the two regions, the genetic variation attributed to their geographical separation was similar to variations observed among greenhouses within individual regions. Fourth*,* depending on the antifungal drug, the frequencies of antifungal resistance often differed between pairs of greenhouses within each of the two regions. Previous studies have shown that greenhouses can be a significant force impacting the genetic variation of fungal pathogens *Botrytis cinerea* and *Fusarium oxysporum* f. sp. *lycopersici* isolated from diseased plants (48, 49). However, the relative contributions of greenhouse separations at a local level to geographically distant populations of these fungal pathogens remained unknown. Using greenhouse populations from two distant regions, our analyses here quantified the relative contributions of both long-distance geographic separation and short-distance physical separation by greenhouses to soil populations of *A. alternata*. The results revealed that greenhouses can facilitate the development of distinct genetic characteristics of *A. alternata* populations among greenhouses located close to each other.

For soil microorganisms, each greenhouse represents a distinct environment where gene flow and genetic exchange among them may be severely limited. Consequently, over time, their gene frequencies and genotype frequencies may diverge from each other, resulting in population differentiation (48). For the soil *A. alternata* population that we examined here, several factors might have contributed to the observed genetic differentiations among the greenhouse populations. First, even though the greenhouses in Shijiazhuang, Hebei province were located close to each other and the natural soil in the region is highly homogeneous, the founding populations of *A. alternata* among greenhouses may differ. Second, greenhouses may grow different crops and vegetables at the same and/or different times. The crops and vegetables themselves and/or their residuals (e.g., fallen leaves and dead roots) and secreted metabolites may differentially impact the growth and reproduction of different genotypes of *A. alternata*. Third, fungicide usage may also differ among greenhouses, contributing to differences in the emergence and reproduction of drug-resistant strains and genotypes among greenhouses. Indeed, the frequencies of antifungal-resistant strains differed among many greenhouses. Further analyses, ideally with data from multiple years, starting from before greenhouses are established, and following with accurate records of cropping systems and fungicide usages, are needed in order to quantify the relative contributions of each of these factors to the observed differentiations among greenhouses.

### Population structure of *A. alternata*

Our STR genotyping of all isolates from the 16 greenhouses in both southwest and northeast China supported that these isolates belonged to three genetically distinct clusters. Among the three clusters, two are widely distributed within both the Kunming and the Shijiazhuang populations. The remaining one was mainly found in the Kunming greenhouse populations in southwest China. Interestingly, within each greenhouse, we found strains belonging to at least two of the three genetic clusters. These results are consistent with historical population divergence within this species in China, with geographic separation playing a significant role in limiting the distribution of clade c strains. Indeed, when both the Shijiazhuang and Kunming populations were included, the Mantel test showed a weak but statistically significant positive correlation between genetic distance and geographic distance among the greenhouse subpopulations of *A. alternata* (*r* = 0.0042, *P* = 0.01). However, within each of the two individual geographic regions, no statistically significant correlation was observed between geographical distances and population genetic distances (21). Such results are consistent with long-distance geographic separation playing an important role in the population structure of *A. alternata* in China. However, between the two regions, evidence for shared ancestry and/or recent gene flow was also found, including the presence of shared alleles and identical or closely related genotypes. Earlier studies have shown that long-distance geographic separation contributes significantly to genetic variations of *A. alternata* isolated from citrus fruits and potatoes (33, 50).

Though the inferred recombination was not random, within each of the three genetic clusters, we found evidence of recombination. In addition, evidence for admixture among the three genetic clusters was also found. Such admixtures could be due to incomplete lineage sorting and/or recent hybridization among the clusters. Indeed, our results suggest that these greenhouse populations of *A. alternata* are evolving rapidly. We also note that the number of genetic clusters may change when more isolates are included for analysis, especially those from geographic regions between Kunming and Shijiazhuang where connecting populations may exist. Analyzing more geographically broadly distributed populations that are separated by short to intermediate distances will help determine the migration patterns and infer potential landscape features impacting the spatial population structure of *A. alternata*.

### Triazole resistance-related mutations at the target *cyp51* gene

Due to the broad use of fungicides in greenhouses for controlling disease caused by microorganisms (51–53), a number of pathogens from greenhouses, such as *Botrytis cinerea,* have shown an increasing resistance to different fungicides (19, 54). A study in 2021 showed that a high frequency (about 80%) of *Aspergillus fumigatus* isolates from nine greenhouses in Kunming showed resistance to at least one triazole drug, and ~30% of the isolates showed cross-resistance to both ITR and VOR (20). The observed triazole resistance in greenhouse populations was much higher than that in natural *A. fumigatus* populations from outdoor areas in Yunnan and other places around the world (55). In this study, we found similar MIC ranges to the four tested fungicides in *A. alternata* from Shijiazhuang greenhouses as the greenhouse populations of *A. alternata* from Kunming (21). Alarmingly, resistance to all four antifungals was found in almost all seven greenhouse populations of *A. alternata* in Shijiazhuang. However, slightly lower frequencies of isolates being resistant to DIF (9.5%), ITR (18.1%), and VOR (17.1%) were found in the Shijiazhuang population than in the Kunming population, where 17.5%, 27.3%, and 26.2% isolates were resistant to DIF, ITR, and VOR, respectively. At present, while the reasons for the different frequencies among greenhouses within and between the two geographic regions are not known, it is tempting to speculate that differences in agriculture fungicide usage were likely a significant contributor. Unfortunately, we were unable to obtain accurate fungicide usage data for the greenhouses in both Kunming and Shijiazhuang. However, around Kunming in southwest China, there is a large fresh-cut flower production business using greenhouses near the Dianchi area, including Jinning county, where greenhouses frequently rotate between growing vegetables and fresh-cut flowers (56). Compared with growing vegetables that have regulations governing pesticide and fungicide usage, there is little regulation in fungicide usage for growing flowers and other ornamental plants (56). Consequently, there was likely an overall higher fungicide usage in greenhouses in Kunming than in Shijiazhuang, creating higher selection pressure and resulting in greater frequencies of fungicide-resistant strains in greenhouses in Kunming than in Shijiazhuang. Similarly, due to differences in susceptibility to fungal infections among vegetables, fungicide usages often differed among vegetables (and by extension, greenhouses) within each of the two regions (20). Together, we believe the observed differences in triazole susceptibilities among greenhouses and between the two geographic regions at least partly reflect their fungicide usage histories.

As a plant pathogenic fungus, *A. alternata* has received significant attention. Indeed, most population studies of *A. alternata* have been on isolates obtained from infected plants. In contrast, despite being one of the most widely distributed saprophytic fungi in soil, relatively little is known about the genetic diversity and antifungal resistance in soil populations of this species. However, the soil population and pathogen population on crops are likely linked, with soil populations serving as reservoirs of infectious particles, while diseased host plants selectively amplify a subset of strains that infected host plants (57). In addition, certain strains could also be selectively amplified by diseased plant tissue residues, such as decaying leaves from previous crops, infecting subsequent crops, generating strains that could potentially cause large-scale disease outbreaks (58). In this and previous studies (20, 21), greenhouses within the same region often had different crop production systems, which in turn could differentially influence pathogen populations, contributing to genetic differentiations among greenhouse populations of *A. alternata*.

More broadly, our analyses showed that many strains of *A. alternata* from these greenhouses are resistant to both clinical and agricultural triazoles. Although the fungus does not represent a direct critical threat to humans at present, infections of humans by *A. alternata* have been reported [e.g., (9)]. Furthermore, in combination with its broad pathogenicity in over 100 plant species (including many crops, fruits, and vegetables) and its ability to produce toxins, its resistance to a range of antifungal drugs presents increasing challenges for disease management in both agriculture and clinics. Indeed, crop plants caused by drug-resistant *A. alternata* can enter the human food chain with the agricultural food products where the pathogen may reproduce, adapt, and cause infection. Further analyses of samples from diverse ecological niches are needed in order to understand how triazole-resistant strains spread in agroecosystems, in the food chain, and in human populations. Such information will be essential for developing sound strategies for controlling and preventing *A. alternata* infections in agricultural environments and in clinical settings (2).

## Data Availability

All data relevant to the conclusions of this study are presented in the manuscript and at GenBank accession numbers PP731575-PP731778. All raw data are available from the author upon request. No high-throughput sequence data were generated during the study.

## SUPPLEMENTAL MATERIAL

Supplemental material spectrum.00390-24-s0001.docx Tables S1-S7; Fig. S1-S7.

ASM does not own the copyrights to Supplemental Material that may be linked to, or accessed through, an article. The authors have granted ASM a non-exclusive, world-wide license to publish the Supplemental Material files. Please contact the corresponding author directly for reuse.
