# Supplementary material for: Greenhouses represent an important evolutionary niche for Alternaria alternata
Source: Microbiol Spectr. 2024 May 10;12(6):e00390-24. doi: 10.1128/spectrum.00390-24 (PMC11237460; doi:10.1128/spectrum.00390-24)
Supplement: Supplemental material — Tables S1-S7; Fig. S1-S7. [file spectrum.00390-24-s0001.docx]

# Supplementary Data

# Greenhouses represent an important evolutionary niche for *Alternaria alternata*

Guangzhu Yang ^a,b,c^, Sai Cui ^a,b^, Wenjing Huang ^c^, Shutong Wang^d^, Jun Ma ^c^#, Ying Zhang ^a^#, Jianping Xu ^a,e,^ #

^a^ State Key Laboratory for Conservation and Utilization of Bio-Resources in Yunnan, Yunnan University, Chenggong District, Kunming, Yunnan, 650500, PRC

^b^ College of Life Science, Yunnan University, Chenggong District, Kunming, Yunnan, 650500, PRC

^c^ Horticultural Research Institute, Yunnan Academy of Agricultural Sciences, Kunming, Yunnan, 650205, PRC

^d^ Hebei Agricultural University, Baoding, Hebei, 071001, PRC

^e^ Department of Biology, McMaster University, Hamilton, Ontario, L8S 4K1, Canada

# Address correspondence to Jun Ma, idyll_001@163.com; Ying Zhang, yingzhang@ynu.edu.cn; Jianping Xu, jpxu@mcmaster.ca

Guangzhu Yang and Sai Cui contributed equally to this work.

Table S1. Information on the seven greenhouse populations of *A. alternata* from Shijiazhuang.

| Greenhouse population | No of isolates | After cloning correction | Operational Years | Crops | Geographical coordinate | |
| --- | --- | --- | --- | --- | --- | --- |
|  |  |  |  |  | Longitude | Latitude |
| SJZ.1 | 41 | 35 | 5 | batata | 114.97 | 37.96 |
| SJZ.2 | 13 | 12 | 3 | tomato | 114.96 | 37.96 |
| SJZ.3 | 29 | 28 | 4 | cucumber | 114.95 | 37.96 |
| SJZ.4 | 38 | 34 | 4 | cucumber | 114.90 | 37.96 |
| SJZ.5 | 40 | 38 | 8 | eggplant | 114.75 | 38.05 |
| SJZ.6 | 36 | 31 | 5 | tomato | 114.74 | 38.05 |
| SJZ.7 | 36 | 23 | 5 | tomato | 114.73 | 38.05 |

Table S2. Number of alleles, private alleles and diversity at each SSR marker in seven greenhouses in Shijiazhuang.

| Pop. | MLG*** | No. of alleles and diversity at each locus (no. of private alleles in parentheses) | | | | | | | | | | | | | | | | | | | | | | |  |
| --- | --- | --- | --- | --- | --- | --- | --- | --- | --- | --- | --- | --- | --- | --- | --- | --- | --- | --- | --- | --- | --- | --- | --- | --- | --- |
|  |  | c10062 | | c9473 | | c10524 | | c9860 | | c3806 | | c10756 | | AEM6 DQ272485 | | AEM9 DQ272486 | | | PAS2 | | | PAS6 | |  |  |
|  |  | NA* | h** | NA | h | NA | h | NA | h | NA | h | NA | h | NA | h | | NA | h | | NA | h | NA | h | NA | |
| SJZ.1 | 35(25) | 3 | 0.297 | 4(1) | 0.532 | 8 | 0.719 | 3 | 0.547 | 5(1) | 0.482 | 5 | 0.458 | 6(1) | 0.726 | | 2 | 0.048 | | 11(2) | 0.844 | 1 | 0 | 48(5) | |
| SJZ.2 | 12(9) | 2 | 0.18 | 2 | 0.26 | 5 | 0.744 | 3 | 0.521 | 2 | 0.26 | 3 | 0.379 | 4 | 0.604 | | 2(1) | 0.142 | | 6(1) | 0.757 | 2 | 0.142 | 31(2) | |
| SJZ.3 | 28(18) | 3 | 0.476 | 3 | 0.447 | 7 | 0.766 | 3 | 0.502 | 5(2) | 0.535 | 5 | 0.521 | 4 | 0.64 | | 2 | 0.128 | | 7 | 0.766 | 2 | 0.128 | 41(2) | |
| SJZ.4 | 34(32) | 3 | 0.555 | 3 | 0.394 | 11(2) | 0.815 | 3 | 0.561 | 6(3) | 0.514 | 4 | 0.281 | 6(2) | 0.67 | | 1 | 0 | | 10 | 0.82 | 3 | 0.148 | 40(7) | |
| SJZ.5 | 38(22) | 2 | 0.26 | 2 | 0.42 | 7 | 0.76 | 3 | 0.464 | 6(2) | 0.561 | 7(1) | 0.64 | 4 | 0.594 | | 2 | 0.095 | | 12(1) | 0.859 | 2 | 0.049 | 47(4) | |
| SJZ.6 | 31(22) | 3 | 0.156 | 2 | 0.486 | 6 | 0.727 | 3 | 0.498 | 6(3) | 0.557 | 4 | 0.295 | 4 | 0.591 | | 1 | 0 | | 11(1) | 0.863 | 2 | 0.105 | 42(4) | |
| SJZ.7 | 23(20) | 3 | 0.549 | 3 | 0.423 | 6 | 0.719 | 3 | 0.545 | 3(1) | 0.364 | 6 | 0.384 | 5 | 0.747 | | 3(1) | 0.202 | | 7(1) | 0.755 | 3 | 0.332 | 42(3) | |
| Total | 171(141) | 3 | 0.353 | 4(1) | 0.423 | 11(2) | 0.750 | 3 | 0.520 | 17(12) | 0.468 | 7(1) | 0.423 | 8(3) | 0.653 | | 5(2) | 0.088 | | 18(6) | 0.809 | 4 | 0.129 | 80(27) | |

*NA: No. of alleles

**h: diversity

***MLG: Multilocus genotype; total number of MLGs (number of private MLGs found only in each greenhouse)

Table S3. Summary results of AMOVA within and among the seven greenhouse populations of *A. alternata* in Shijiazhuang.

| Source | df | SS | MS | Est. Var. | % | AMOVA Statistics | Value | P |
| --- | --- | --- | --- | --- | --- | --- | --- | --- |
| Among Greenhouses | 6 | 27.982 | 4.664 | 0.068 | 3%* | PhiPT | 0.027 | 0.001 |
| Within Greenhouses | 226 | 550.834 | 2.437 | 2.437 | 97%* | Nm | 17.997 |  |
| Total | 232 | 578.815 |  | 2.505 | 100% |  |  |  |

*, P<0.05

Table S4. Summary results of AMOVA within and among the seven greenhouse populations in Shijiazhuang based on clone-corrected samples.

| Source | df | SS | MS | Est. Var. | % | AMOVA Statistics | Value | P |
| --- | --- | --- | --- | --- | --- | --- | --- | --- |
| Among Greenhouses | 6 | 19.664 | 3.277 | 0.029 | 1%* | PhiPT | 0.012 | 0.026 |
| Within Greenhouses | 194 | 477.226 | 2.460 | 2.460 | 99%* | Nm | 42.617 |  |
| Total | 200 | 496.891 |  | 2.489 | 100% |  |  |  |

*, P<0.05

Table S5. Pairwise differentiations among 16 greenhouse populations of *A. alternata* from two geographic regions in China.

|  | SJZ.1 | SJZ.2 | SJZ.3 | SJZ.4 | SJZ.5 | SJZ.6 | SJZ.7 | YN.1 | YN.2 | YN.3 | YN.4 | YN.5 | YN.6 | YN.7 | YN.8 | YN.9 |
| --- | --- | --- | --- | --- | --- | --- | --- | --- | --- | --- | --- | --- | --- | --- | --- | --- |
| SJZ.1 | 0.000 | 0.100 | 0.108 | 0.002 | 0.029 | 0.310 | 0.188 | 0.001 | 0.001 | 0.001 | 0.001 | 0.002 | 0.001 | 0.001 | 0.001 | 0.001 |
| SJZ.2 | 0.024 | 0.000 | 0.426 | 0.419 | 0.444 | 0.201 | 0.342 | 0.002 | 0.178 | 0.005 | 0.008 | 0.339 | 0.025 | 0.001 | 0.008 | 0.007 |
| SJZ.3 | 0.014 | 0.000 | 0.000 | 0.372 | 0.443 | 0.161 | 0.406 | 0.001 | 0.057 | 0.001 | 0.002 | 0.080 | 0.005 | 0.001 | 0.003 | 0.001 |
| SJZ.4 | 0.043 | 0.000 | 0.001 | 0.000 | 0.104 | 0.008 | 0.155 | 0.001 | 0.004 | 0.001 | 0.001 | 0.016 | 0.001 | 0.001 | 0.001 | 0.001 |
| SJZ.5 | 0.023 | 0.000 | 0.000 | 0.012 | 0.000 | 0.145 | 0.218 | 0.001 | 0.004 | 0.001 | 0.001 | 0.011 | 0.001 | 0.001 | 0.001 | 0.001 |
| SJZ.6 | 0.004 | 0.015 | 0.011 | 0.040 | 0.010 | 0.000 | 0.175 | 0.001 | 0.001 | 0.001 | 0.001 | 0.001 | 0.001 | 0.001 | 0.001 | 0.001 |
| SJZ.7 | 0.009 | 0.006 | 0.000 | 0.012 | 0.007 | 0.011 | 0.000 | 0.001 | 0.086 | 0.001 | 0.001 | 0.160 | 0.023 | 0.001 | 0.001 | 0.001 |
| YN.1 | 0.188 | 0.134 | 0.139 | 0.157 | 0.168 | 0.200 | 0.130 | 0.000 | 0.228 | 0.374 | 0.164 | 0.004 | 0.043 | 0.024 | 0.001 | 0.357 |
| YN.2 | 0.073 | 0.017 | 0.023 | 0.045 | 0.058 | 0.073 | 0.021 | 0.013 | 0.000 | 0.103 | 0.420 | 0.435 | 0.313 | 0.029 | 0.001 | 0.326 |
| YN.3 | 0.216 | 0.162 | 0.173 | 0.184 | 0.200 | 0.236 | 0.166 | 0.000 | 0.037 | 0.000 | 0.049 | 0.006 | 0.028 | 0.016 | 0.002 | 0.381 |
| YN.4 | 0.093 | 0.070 | 0.067 | 0.082 | 0.092 | 0.104 | 0.065 | 0.019 | 0.000 | 0.050 | 0.000 | 0.069 | 0.031 | 0.003 | 0.014 | 0.144 |
| YN.5 | 0.047 | 0.006 | 0.016 | 0.029 | 0.032 | 0.051 | 0.012 | 0.072 | 0.000 | 0.102 | 0.025 | 0.000 | 0.278 | 0.001 | 0.004 | 0.048 |
| YN.6 | 0.100 | 0.051 | 0.049 | 0.067 | 0.069 | 0.092 | 0.040 | 0.047 | 0.003 | 0.073 | 0.041 | 0.005 | 0.000 | 0.002 | 0.005 | 0.159 |
| YN.7 | 0.165 | 0.125 | 0.131 | 0.160 | 0.155 | 0.176 | 0.126 | 0.046 | 0.037 | 0.058 | 0.066 | 0.067 | 0.068 | 0.000 | 0.001 | 0.014 |
| YN.8 | 0.118 | 0.134 | 0.108 | 0.135 | 0.125 | 0.150 | 0.121 | 0.138 | 0.111 | 0.150 | 0.103 | 0.073 | 0.104 | 0.140 | 0.000 | 0.003 |
| YN.9 | 0.116 | 0.083 | 0.089 | 0.103 | 0.104 | 0.127 | 0.079 | 0.000 | 0.004 | 0.000 | 0.016 | 0.026 | 0.011 | 0.041 | 0.103 | 0.000 |

Values at bottom left diagonal represent pairwise Fst while those at top right diagonal represent p values.

Table S6. Summary results of AMOVA among greenhouse populations of *A. alternata* from two different regions in China.

| Source | df | SS | MS | Est. Var. | % | AMOVA Statistics | Value | P |
| --- | --- | --- | --- | --- | --- | --- | --- | --- |
| Among regions | 1 | 51.388 | 51.388 | 0.234 | 7% | PhiPT | 0.103 | 0.001 |
| Among greenhouses | 14 | 73.181 | 5.227 | 0.097 | 3% |  |  |  |
| Within Greenhouses | 377 | 1085.194 | 2.878 | 2.878 | 90% | Nm | 4.352 |  |
| Total | 392 | 1209.763 |  | 3.209 | 100% |  |  |  |

Table S7. Pearson correlation coefficients between MIC values among the four tested triazoles in the total greenhouse sample from Shijiazhuang.

|  | Pearson correlation coefficient of *A. alternata*: | | | |
| --- | --- | --- | --- | --- |
|  | MICs to triazole | | | |
|  | ITR | VOR | TEB | DIF |
| ITR | 0 |  |  |  |
| VOR | 0.297^**^ | 0 |  |  |
| TEB | 0.399^**^ | 0.503^**^ | 0 |  |
| DIF | 0.366^**^ | 0.418^**^ | 0.382^**^ | 0 |

**, P<0.05


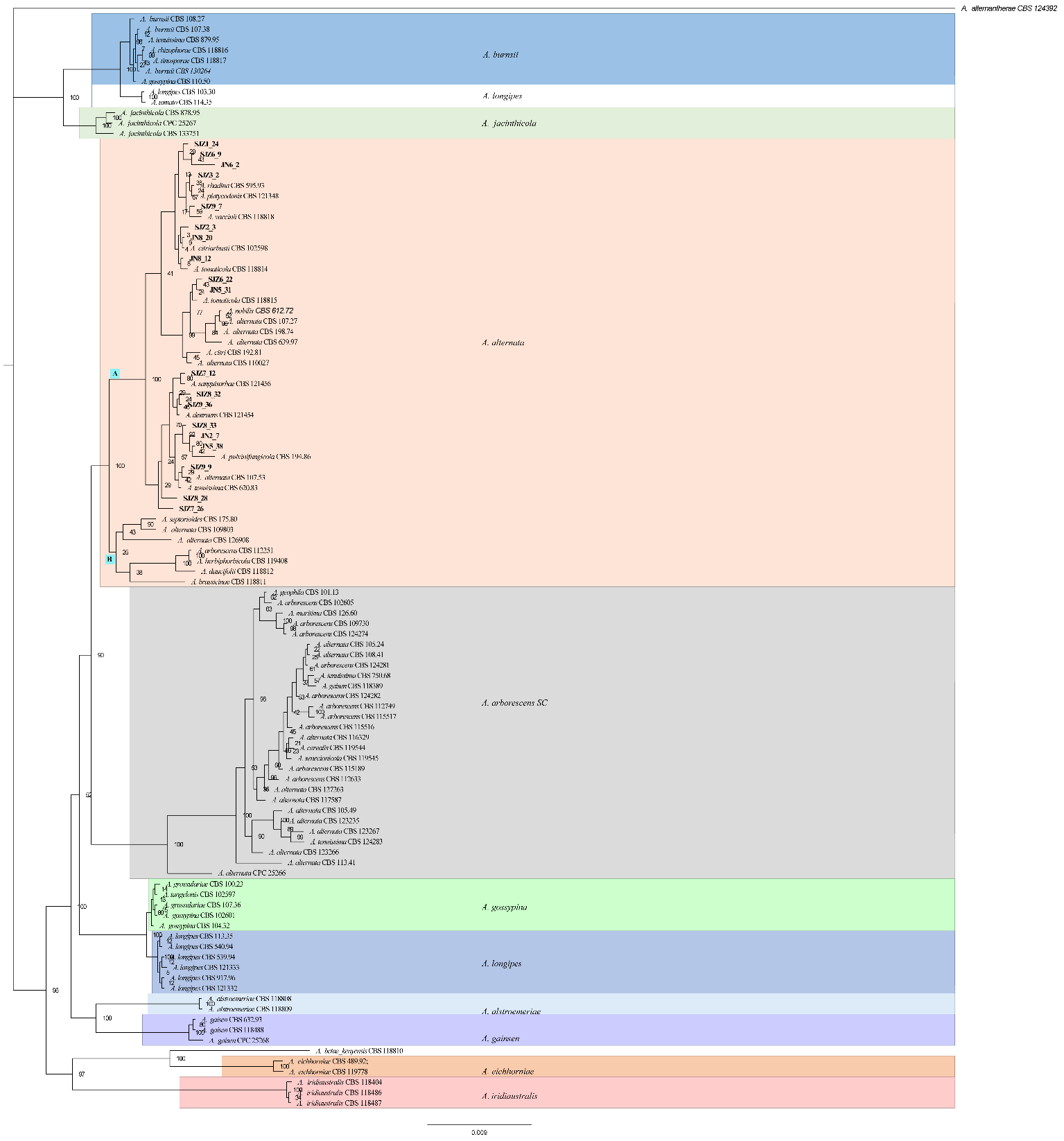


Figure S1. Bayesian consensus tree based on the concatenated DNA sequences at the ITS, *gapdh*, t*ef1*, *rpb2*, *Alta1*, *endoPG* and OPA10-2 gene loci among 90 *Alternaria* strains, including thirteen representative strains from our greenhouse populations. The phylogenetic analysis confirmed that our strains belonged to *A. alternata* sensu stricto. The tree was rooted with *A. alternantherae* strain CBS 124392.


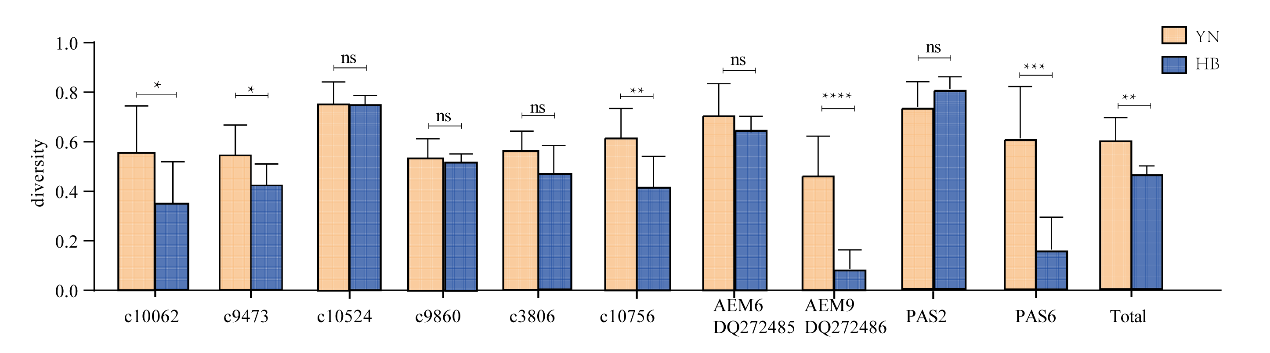


Figure S2. t-test of difference of each STR loci between Kunming and Shijiazhuang populations

Figure S3. Mantel test of the relationship between the frequency difference of private genotypes for the pairs of greenhouses and geographical distance (GGD) among the seven greenhouses in Shijiazhuang.

Figure S4. Mantel test of the relationship between the frequency difference of private genotypes for the pairs of greenhouses and geographical distance (GGD) among the seven greenhouses in Yunnan.


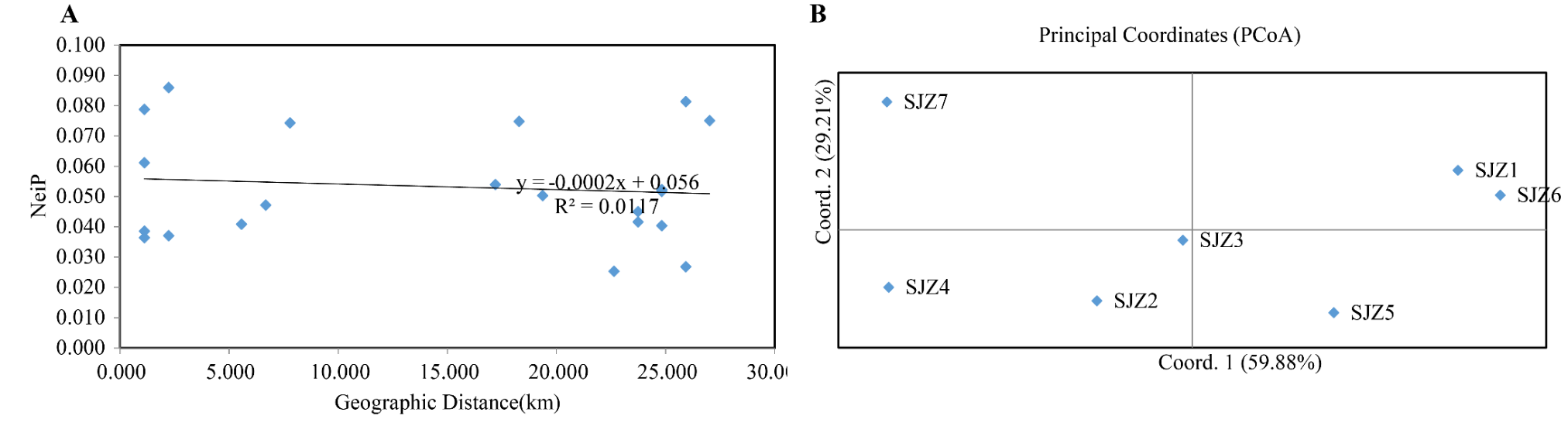


Figure S5. Mantel test of the relationship between Nei’s genetic distance (NeiP) for ten microsatellite markers and geographical distance (GGD) among the seven greenhouse populations of *A. alternata*.

Figure S6. Mantel test of the relationship between Nei’s genetic distance (NeiP) for ten microsatellite markers and geographical distance (GGD) among the 16 *A. alternata* greenhouse populations in Kunming and Shijiazhuang.


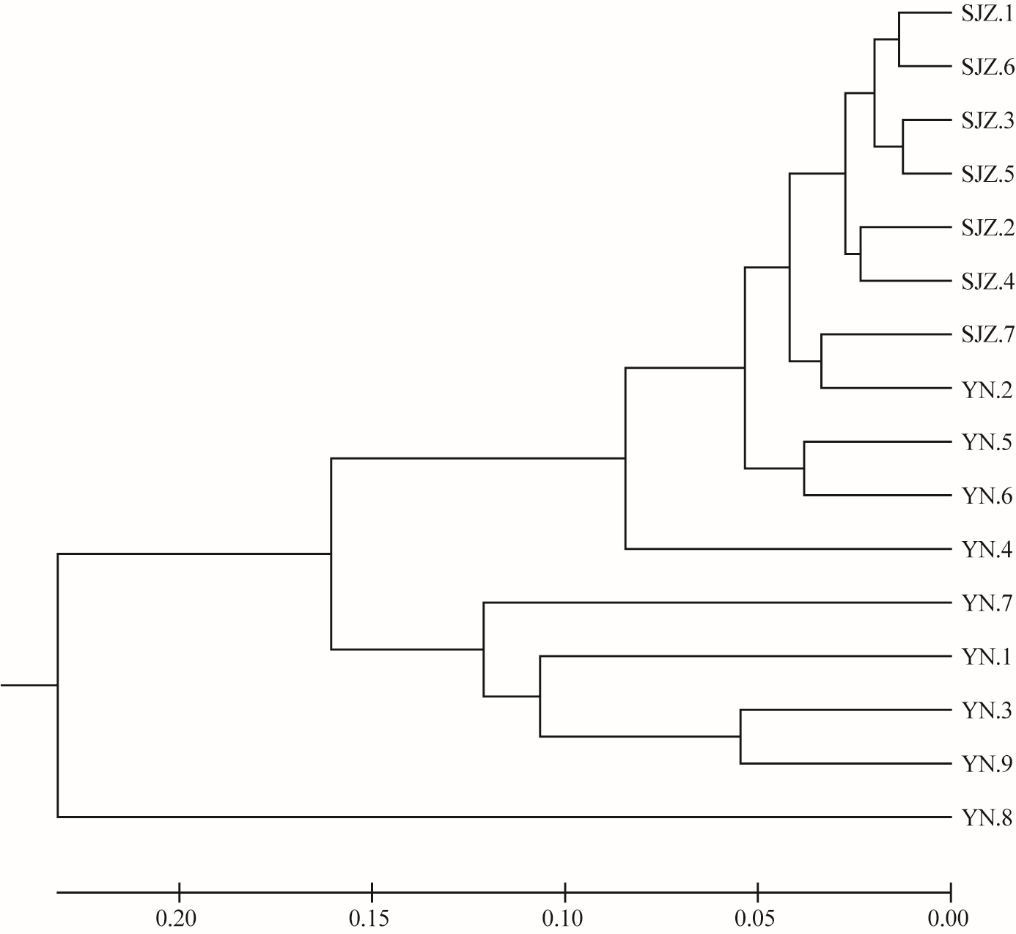


Figure S7. Unweighted pair-group method with arithmetic mean (UPGMA) dendrogram showing genetic relationships among the 16 greenhouse populations of *Alternaria alternata* sampled from Kunming and Shijiazhuang based on Nei’s unbiased genetic distance over 1000 replicates.
